# Characterization of quasispecies of severe fever with thrombocytopenia syndrome virus

**DOI:** 10.1128/jvi.01794-24

**Published:** 2025-04-09

**Authors:** Sithumini M. W. Lokupathirage, Devinda S. Muthusinghe, Rakiiya S. Sarii, Olusola A. Akanbi, Kenta Shimizu, Yoshimi Tsuda, Kumiko Yoshimatsu

**Affiliations:** 1 Graduate School of Infectious Diseases, Hokkaido University 592521, Sapporo, Japan; 2 Institute for Genetic Medicine, Hokkaido University 89291, Sapporo, Japan; 3 National Research Center for the Control and Prevention of Infectious Diseases, Nagasaki University 12961, Nagasaki, Japan; 4 Noguchi Memorial Institute for Medical Research 118922, Accra, Ghana; 5 Center for Disease Control and Prevention, Abuja, Nigeria; 6 Graduate School of Medicine, Gunma University, Maebashi, Japan; Lerner Research Institute, Cleveland Clinic, Cleveland, Ohio, USA

**Keywords:** SFTSV, CPE, low-pH-dependent cell fusion, cell death

## Abstract

Three specific amino acid variations have been identified in the quasispecies of the isolated YG1 strain of severe fever with thrombocytopenia syndrome virus (SFTSV): Gn (Y328H), Gc (R624W), and L (N1891K). The Gn (Y328H) accounted for 26.9% of the viruses in the patient’s blood. The other two mutations are less frequent, indicating that these mutations appeared during propagation in Vero E6 cells. To investigate the effects of each mutation on viral properties, we evaluated viruses with one to three mutations. Mutations Y328H and R624W in glycoprotein (GP) resulted in increased plaque size and cell fusion activity. Viruses with the N1891K mutation in L showed a notable cytopathic effect (CPE), which was inhibited by a pan-caspase inhibitor, suggesting that caspase-dependent cell death occurred. Programmed cell death-associated caspases were induced in both CPE-inducing and wild-type virus-infected cells. Furthermore, infection with the wild-type virus suppressed actinomycin D-induced cell death. These results suggest that SFTSV-infected cells initiate programmed cell death, whereas the wild-type virus inhibits cell death. Additionally, the recombinant single mutant virus outcompeted by a 10-fold lower amount of the wild-type virus in Vero E6 cells, indicating that the mutations were not advantageous for viral propagation in Vero E6 cells. These findings suggest that the quasispecies composition of SFTSV is influenced by the propagative environment.

**IMPORTANCE:**

This study presents findings on viral pathogenesis by analyzing quasispecies derived from a fatal case of severe fever with thrombocytopenia syndrome virus (SFTSV) infection. Analysis of recombinant SFTSV with mutations in Gn and Gc suggested that combinations of mutations may enhance the viability of mutant viruses, thereby selecting viruses to create a suitable population for propagation. The N1891K mutation in the L protein of SFTSV is associated with promoting cytopathic effects (CPE). Conversely, the wild-type virus, which is the predominant virus in infected patients, suppresses cell death. It has been suggested that SFTSV possesses a mechanism to evade cell death for prolonged viral propagation within the infected cells. Although the precise mechanism remains unknown, RNA virus polymerase may be involved in regulating cell death. This study contributes to our understanding of the mechanisms underlying the adaptation and survival of viruses as quasispecies.

## INTRODUCTION

Severe fever with thrombocytopenia syndrome (SFTS) is caused by the SFTS virus (SFTSV), a member of the order *Hareavirales* and family *Phenuiviridae*. The SFTS is widely distributed in East Asian countries, including China, Japan, South Korea, and Vietnam (1–4). In addition, this virus has also been detected in several other countries, such as Myanmar and Pakistan (5, 6). The mortality rate of SFTS is as high as 30% in Japan, and it is a public health concern in other countries as well (7–10). The SFTSV YG1 strain was isolated from the first patient with SFTSV in Yamaguchi Prefecture, Japan (2).

SFTSV consists of three negative-sense segments, designated as small (S), medium (M), and large (L). The S segment is an ambisense RNA encoding a nucleocapsid protein and a non-structural protein (11); the M segment encodes two envelope glycoproteins (GP), Gn and Gc; and the L segment encodes an L protein, which is an RNA-dependent RNA polymerase (RdRp).

In viruses of the family *Phenuiviridae*, entry into host cells is initiated by binding of GPs (Gn and Gc) to cell surface receptors, with endocytosis occurring in a receptor-mediated manner (12–15). Following endocytosis, viral GPs fuse with the endosomal membrane under acidic conditions, causing low pH-dependent cell fusion in infected cells (16–18). Structural models of SFTSV Gn and Gc show that some amino acid residues are crucial for low pH-dependent cell fusion and syncytial formation (19, 20). Following cell entry, the cells infected with Phenuiviruses, such as SFTSV and the Rift Valley fever virus, can cause cytopathic effect (CPE) (21, 22). Gao et al. reported that the cell death induced in human microglial HMC3 cells by SFTSV infection was the result of NOD-like receptor protein 3 inflammasome activation, which led to the secretion of interleukin (IL)-1β and pyroptosis (23). Whereas, it is known that most cell lines and primary cultures are not killed by SFTSV infection. Suzuki et al. reported that B cells are the target cells for lethal SFTSV infection (24). Transformed B cells and the primary culture of peripheral B cells did not die from SFTSV infection. Vero E6 and Huh7 cells used for virus isolation also did not show CPE. Thus, the relationship between SFTSV infection and cell death remains largely unexplored.

In a previous study, we established three subclones (E3, A4, and B7) from the YG1 strain using the limiting dilution method with pH-dependent cell fusion activity and CPE (21). The genome of subclone E3 was identical to that of the parental YG1 strain, whereas subclones A4 and B7 had two amino acid mutations in glycoproteins Gn (Y328H) and Gc (R624W). Subclones A4 and B7 were selected for intense cell fusion activity under acidic conditions. Subclone B7 had another amino acid mutation, N1891K, in its L protein. In addition, B7 (Gn: Y328H, Gc: R624W, and RdRp: N1891K) showed robust CPE, indicative of the involvement of L protein N1891K in the difference in inducing CPE between the subclones (21). In addition, the results of a minigenome assay indicated that the polarization of the amino acid at position 1891 of the L protein is critical for its function, particularly its polymerase activity (25). However, no substantial difference was observed in the *in vitro* replication of the virus.

Next-generation sequencing revealed that the Gn: Y328H mutation accounts for 26.9% of the viral population in the blood of the patient with YG1 strain infection (2, 26). However, after isolating the YG1 strain, the rate of Gn: Y328H mutation decreased to approximately 10%. In contrast, the other two mutations, Gc: R624W and L: N1891K, appeared infrequently in the patient’s blood and the isolated YG1 strain. The roles of these mutations in the virological characteristics and population structure of SFTSV remain unclear. Understanding the emergence and survival of viruses whose pathogenicity changes due to mutations is essential for elucidating their pathogenicity.

In this study, a panel of recombinant viruses bearing mutations was developed using a reverse genetics system (11) with the aim of comparing the unique virological characteristics of each mutation. In addition, their effects on quasispecies were evaluated, and the impact of these mutations on quasispecies is discussed.

## MATERIALS AND METHODS

### Cells and viruses

Vero E6 cells (ATCC C1008) were maintained in Eagle’s minimum essential medium (Gibco; Thermo Fisher Scientific, MA, USA) supplemented with 5% heat-inactivated fetal bovine serum (FBS) (Biowest, Nuaillé, France), 1% MEM non-essential amino acids (Gibco), 1% insulin-transferrin-selenium (Gibco), 1% penicillin (50 units/mL), streptomycin (50 µg/mL; Sigma-Aldrich Co., St Louis, MO, USA), and 1% gentamicin (100 µg/mL; Sigma-Aldrich). BSR-T7/5 cells stably expressing T7 RNA polymerase were kindly provided by Dr. K. K. Conzelmann (Max-von-Pettenkofer Institute, Munich, Germany) (27). The cells were maintained in low-glucose Dulbecco’s modified Eagle medium (Sigma-Aldrich) supplemented with 10% FBS, 10% tryptose phosphate broth (Gibco), and antibiotics (1 mg/mL geneticin [G418] [Nacalai Tesque, Kyoto, Japan] or 1% penicillin and streptomycin). Huh7 human hepatoma and 293T cells (Riken, Japan) were grown and maintained in Dulbecco’s modified Eagle medium (Thermo Fisher Scientific) supplemented with 10% FBS and 1% penicillin-streptomycin. All cells were cultured in a 5% CO_2_ incubator at 37°C. The SFTSV subclones of the YG1 strain, A4, E3, and B7, were used as controls (21). The viral infection experiments were performed in a biosafety level 3 (BSL-3) facility at the Institute for Genetic Medicine, Hokkaido University.

### Plasmids

Full-length YG1 S, M, and L segment constructs were generated by cloning into TVT7R (0,0), kindly provided by Dr. Benjamin Brennan, Glasgow Center for Virus Research, Scotland, United Kingdom (11). Cloning was conducted using an In-Fusion HD cloning kit (Takara Bio, CA, USA), and the resulting plasmids were named pTVT7_YG1_S, pTVT7_YG1_M, and pTVT7_YG1_L. The amino acid mutation Y328H was introduced into the pTVT7_YG1_M construct using site-directed mutagenesis using the KOD One PCR Master Mix (Toyobo, Osaka, Japan) and primers containing the mutation (forward: CGTGTCAGACCAAAATGCCATGGTTTCTCCAGAATGA, and reverse: TCATTCTGGAGAAACCATGGCATTTTGGTCTGACACG). The mutation N1891K was introduced to the pTVT7_YG1_L construct, and R624W was introduced to pTVT7_YG1_M using the In-Fusion HD cloning kit (Takara Bio); primers containing mutation for insert sequence and primers containing mutation for vector sequence (AACTTGGAAGTGC-TTTGTGGTAGG and CAATTGGGCCTGGTCACATGCCTCAGTTC for the mutation N1891K, or GACCAGGCCCAATTGTCAAGAGTTTTC and AAGCACTTCCAAGT-TCATCTGGGCGTCT for the mutation R624W).

### Recombinant virus generation

Eight different recombinant viruses were generated by transfecting 1.6 × 10^6^ cells of BSR-T7/5 cells with 2 µg of each pTVT-based plasmids expressing viral genomic segments with or without point mutations, 0.2 µg pTM1-HB29ppL and 1 µg pTM1-HB29N (11), kindly provided by Dr. Benjamin Brennan. Transfection experiments were conducted using TransIT-LT1 reagent (Mirus Bio LLC, Madison, WI, USA). After 5 days of incubation, the recombinant virus supernatant was harvested as P1. The P1 supernatant was then blindly passed into Vero E6 cells, and the recombinant virus stock P2 was prepared by harvesting the culture medium 7–9 days post-infection (dpi), depending on the CPE. To confirm virus rescue, total RNA was extracted from the P2 virus using Isogen LS (Nippon Gene) according to the manufacturer’s protocol, and cDNA synthesis was performed using the ReverTra Ace qPCR RT Master Mix (Toyobo), and real-time PCR was carried out using KAPA SYBR FAST qPCR kit (KAPA Biosystems, Wilmington, MA, USA) and primers SFTSV-L-3613F (AGCAGCGTCTCACCAAATCTC) and SFTSV-L-3730R (GCAGGAGCTGAGCGCACTGT).

Each P2 virus was inoculated into Vero E6 cells for viral amplification, and the seed virus (P3) was obtained after 6 days of incubation. The eight rescued viruses were named after their mutations for ease of application (Table 1). To confirm the genome sequence of recombinant viruses, total RNA was extracted from the P2 inoculated cells that produced P3 viruses using Isogen (Nippon Gene, Tokyo, Japan). After cDNA synthesis using the ReverTra Ace reverse transcriptase (Toyobo), 12 PCR products covering the entire viral genome were amplified by Quick Taq HS DyeMix (Toyobo). The nucleotide sequences of the PCR products were confirmed using both an amplicon-based ligation sequencing protocol with a native barcoding kit on a MinION device (SQK-LSK1-9 and EXP-NDB104; Oxford Nanopore Technologies, Oxford, UK) and Sanger sequencing.

**TABLE 1:** Recombinant viruses and the respective plasmid constructs used for the reverse genetics system^*a*^^,^^*b*^

Recombinant viruses	S genome	M genome: aa 328/624 (Gn/Gc)				L genome: aa 1891		Titer (PFU/mL)
	(Wild type)	Y/R (Wild type)	H/R	Y/W	H/W	N (Wild type)	K	
rYG1 (parent)	+	+	−	−	−	+	−	2.2 × 10^6^
rGn	+	−	+	−	−	+	−	1.8 × 10^6^
rGc	+	−	−	+	−	+	−	1.3 × 10^7^
rL	+	+	−	−	−	−	+	9.0 × 10^6^
rGn/L	+	−	+	−	−	−	+	1.2 × 10^6^
rGc/L	+	−	−	+	−	−	+	1.5 × 10^7^
rGn/Gc	+	−	−	−	+	+	−	6.0 × 10^6^
rGn/Gc/L	+	−	−	−	+	−	+	2.8 × 10^4^

^*a*^ +, a combination of amino acid sequences in the recombinant virus; −, amino acid sequence not used.

^*b*^ (wild type), amino acid sequence identified in the parent YG1.

### Plaque assay and virus titration

Vero E6 cells were seeded at 4 × 10^5^ cells/mL in a 6-well plate, infected with serial dilutions of each recombinant virus, and cultured in an overlay medium: MEM containing 0.8% SeaKem ME agarose (Takara Bio) and 4% FBS. After 7 dpi, 2 mL of neutral red solution (0.1 mg/mL in MEM; Sigma-Aldrich) was added to the overlay medium. After incubation for 24 h, viral foci were visible. The number and diameter of the foci were evaluated by using the open-source Fiji/ImageJ software (https://imagej.net). The titer of each recombinant virus was calculated from the number of foci in suitable dilution and determined as plaque-forming units (PFU). The plaque diameter data were analyzed by using Ordinary one-way analysis of variance (ANOVA) followed by Tukey’s multiple comparison analysis in GraphPad Prism version 10.3.1 (464) for macOS (GraphPad Software, Boston, Massachusetts USA, www.graphpad.com).

### Cell fusion assay

Vero E6 cells were seeded to a 96-well plate inoculated with serial dilutions of each recombinant virus and incubated for 7 days at 37°C. The cells were fixed with Mildform 10 NM, a 10% formalin neutral buffer-methanol solution (Wako Pure Chemical Corporation, Osaka, Japan) for 20 min and stained with azur-eosin-methylene blue solution (Muto Pure Chemicals Co., Ltd., Tokyo, Japan) to visualize cell fusion. Images were acquired using a Keyence BZ-X 800 microscope (Keyence Corporation, Osaka, Japan).

### Pseudotype virus assay

The mammalian expression vector pCAGGS-YG1-GP and its mutants have been cloned previously (20). The pseudotype vesicular stomatitis virus (VSV) possessing luciferase genes was kindly provided by Dr. Heinz Feldmann (DIS, NIAID, NIH). Each pseudotyped VSV was generated as previously reported. Briefly, 293T cells were transfected with each plasmid vector, pCAG-GP-Y/R, pCAG-GP-H/R, pCAG-GP-Y/W, and pCAG-GP-H/W, using TransIT-LT1 and incubated for 30 h at 37°C. Pseudotype VSV possessing VSV-G was inoculated, and the culture supernatant was collected 13 h after inoculation. The VSV particles produced in the collected supernatants were evaluated by western blot analysis using an anti-VSV M monoclonal antibody (kindly provided by Professor Ayato Takada, Hokkaido University, Japan). Vero E6 cells were inoculated with 10 times dilution of each VSV∆G/SFTSV (GP-Y/R, GP-H/R, GP-Y/W, or GP-H/W) and incubated for 18 h at 37°C. Luciferase activity in the cell lysates was determined using the steady-Glo luciferase assay system (Promega, Madison, WI, USA). One-way ordinary ANOVA analysis was conducted to compare the luciferase activity.

### Treatments of a pan-caspase inhibitor for rSFTSV-infected cells

Vero E6 cells were inoculated with rGn/Gc/L, rL, and rYG1, and at 3 dpi, media were replaced with fresh media containing 50 µM of pan-caspase inhibitor Z-VAD-FMK (Selleck chemicals, TX, USA). Dimethyl sulfoxide (Sigma-Aldrich) was used as a diluent for the pan-caspase inhibitors. Mock-infected Vero E6 cells were used as the controls. At 5 dpi, the cells were fixed with Mildform and stained with Giemsa azure-eosin-methylene blue solution for visualization.

### Western blot analysis

The recombinant viruses, rYG1 and rL were inoculated into Vero E6 cells in a 6-well plate at a multiplicity of infection of 0.05. At 1, 4, and 7 dpi, the cells were collected using a cell scraper and rinsed once with ice-cold phosphate-buffered saline, followed by lysis with 200 µL of sample buffer solution with 2-mercaptoethanol (Fujifilm-Wako) and incubated at 100°C for 5  min. The cell lysates were loaded onto a sodium dodecyl-sulfate-polyacrylamide gel electrophoresis gel (e-PAGEL 1020L, Atto, Tokyo, Japan) and electroblotted onto a 0.45 µm pore immunoblot polyvinylidene fluoride membrane. The membranes were incubated with primary antibodies: anti-caspase 1, anti-caspase 3 (Cell Signaling, Danvers, MA, USA), and anti-SFTSV N protein (28) for 1 h at room temperature or overnight at 4°C. Horseradish peroxidase-conjugated mouse anti-rabbit IgG (Cell Signaling) was used as the secondary antibody and incubated for 1 h at room temperature. Bound antibodies reacted with Pierce ECL Plus Western Blotting Substrate (Thermo Fisher Scientific) and were detected using Lumino Graph 1 (Atto). An HRP-conjugated anti-glyceraldehyde-3-phosphate dehydrogenase (GAPDH) antibody (Cell Signaling) was used to detect GAPDH as the control protein in this experiment.

### Cell death inhibition by the rYG1 infection

Actinomycin D (Sigma-Aldrich), an inducer of apoptosis, in high to low concentrations (500, 400, 300, 200, and 100 ng/µL) were mixed with equal volumes of rYG1 with 10 PFU/well and inoculated into the monolayer of Vero E6, Huh-7, and BHK/T7-9 cells. rYG1 infection and actinomycin D stimulation alone were used as controls. After 3 dpi, the cells were fixed and stained as described above.

### Co-infection of recombinant viruses

The rYG1 was inoculated into Vero E6 cells at a multiplicity of infection of 0.2 together with rGn, rGc, and rL viruses (ratios of 10:1, 1:1, or 0.1:1 calculated from FFU of each virus stock) in a monolayer 6-well plate. Eight days after inoculation, the culture supernatant was collected. Total RNA was extracted from the culture supernatant using Isogen LS, and cDNA was produced using a ReverTra Ace cDNA synthesis kit. Three parts of the genome, those were M genome segment 804–1325, 1792–2381, and L genome segment 5135–5883 were amplified using the KOD One PCR Master Mix (Toyobo, Osaka, Japan). PCR products were sequenced using MinION, as described above. The ratio of wild type to mutant at 1,000 and 1,888 in the M genome segment and 5,689 in the L genome segment were estimated from the percentage of mutants in sequence reads analyzed by GENETYX-NGS/MAC software (Nihon Server, Tokyo Japan).

## RESULTS

### Establishment of recombinant viruses using a reverse genetics system

We established a reverse genetics system for the SFTSV-YG1 strain and rescued recombinant viruses from the original YG1 strain with single, double, or triple mutations. Together with helper plasmids, sets of plasmids were transfected into BSR-T7/5 cells (Table 1). The supernatant was harvested and designated as P1. P1 was blindly inoculated into Vero E6 cells for expansion and collected supernatants as P2. To detect the production of the respective recombinant viruses, P2 was used for genome detection by real-time PCR and indirect immunofluorescence antibody tests (data not shown). Further passaging was conducted using Vero E6 cells to obtain the virus as P3, which was used as the seed virus for downstream analysis. The eight rescued viruses were designated as rYG1, rGn, rGc, rL, rGn/L, rGc/L, rGn/Gc, or rGn/Gc/L depending on the mutation(s). All recombinant viruses, except rYG1, showed plaques, with virus titers higher than 10^6^ PFU/mL, except for rGn/Gc/L (Table 1). The nucleotide sequences of all recombinant viruses from P3 produced cells were confirmed using MinION and Sanger sequencing before subsequent experiments (data not shown).

### Plaque formation and titers of recombinant viruses

**Fig 1:**
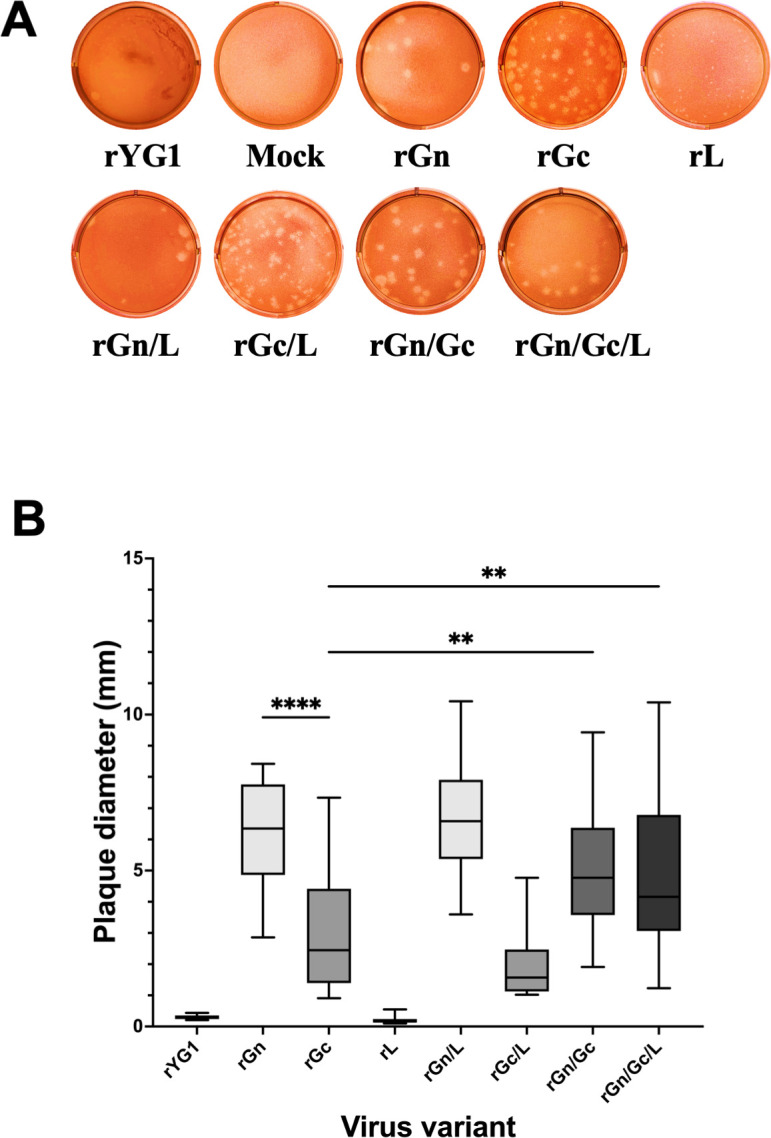
Plaque-forming activity of recombinant viruses. (A) Plaques of the recombinant viruses. Plaque photographs were taken 10 days after inoculation and 3 days after the overlay of neutral red, except for rGc and rGn/Gc, which were taken at 9 dpi. (B) Size of plaques. The plaque diameters were measured. Box-and-whisker plots show the diameter of randomly selected 25 plaques of each recombinant virus where whiskers represent minimum to maximum data points (corresponding to **P ≤ 0.01, ****P ≤ 0.0001).

In a previous study, we demonstrated that subclone E3, possessing an identical sequence to the dominant sequence of the YG1 strain, did not show clear plaques, indicating that rYG1 possesses characteristics similar to those of subclone E3. The plaques formed by the recombinant viruses revealed different phenotypes depending on mutations in the virus (Fig. 1A). Viral plaques caused by rGn have a clear, smooth, and large appearance, whereas those caused by rGc have a rough, smoky appearance. Gn and Gc mutations increased the plaque size (Fig. S1). In particular, the rGn mutant produced the largest size plaques. In addition, the Gc mutant together with the Gn mutation significantly increased plaque size compared to rGc, suggesting that the Gn mutation has a significant effect on increasing the plaque size (Fig. 1B). The results of the statistical analysis comparing the plaque diameter of the various viruses are shown in Fig. S1. rGc also formed visible plaques; however, no increase in plaque size was observed as a consequence of the introduction of the Gc mutation in addition to the Gn mutation. In contrast, rL plaques appeared as pinpricks. The plaques of rYG1 were similar in size to those of rL; however, they were very smoky, making them difficult to see clearly in the picture. Interestingly, the cells in the rL plaque had died. With the addition of the L mutation, the cells in the plaque were killed, resulting in a transparent plaque. The rGn/Gc/L formed a well-defined plaque, similar to that of rGn.

### Assessment of low pH-dependent cell fusion activity and CPE of recombinant viruses

**Fig 2:**
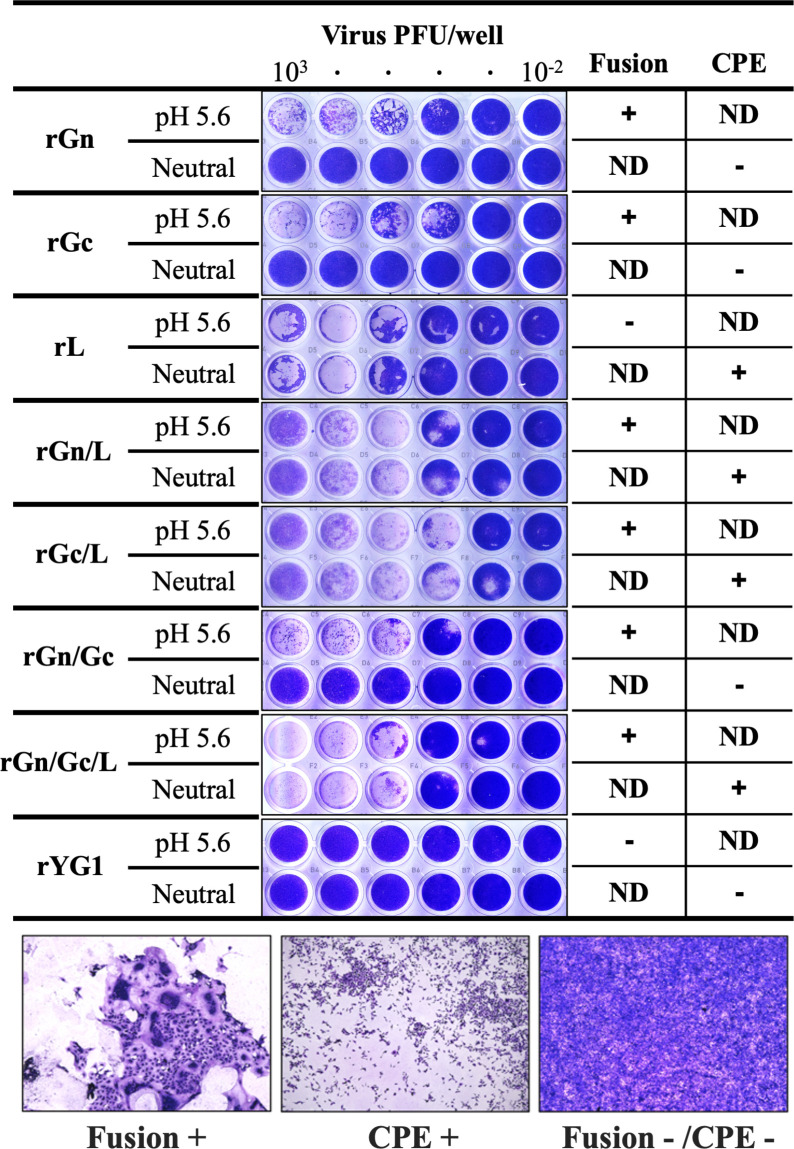
Assessment of low pH-dependent cell fusion activity and CPE of recombinant viruses. Vero E6 cells were inoculated with serial dilution of each recombinant virus and incubated for 7 days at 37°C. The medium was replaced with 50 mM acetate-buffered saline (pH 5.6), and the cells were incubated for 2 min at room temperature. Then, the acetate buffer was replaced with fresh growth medium and incubated for 24 h at 37°C, 5% CO_2_. The wells in the upper panels show Giemsa staining results after low-pH treatment. Wells in the lower panels show Giemsa staining results without low pH treatment. Examples of the evaluations are shown below (+, positive; − negative; ND, not determined).

We previously demonstrated that subclone B7 only demonstrated robust CPE in infected cells, and a mutation in R624W in Gc was responsible for strong syncytia formation under low pH conditions (20, 21). The recombinant virus rGc showed strong syncytial formation under low pH conditions, as confirmed by the protein expression assay. In contrast to previous GP expression assays, rGn showed weak cell fusion activity under low-pH conditions. Neither rGn nor rGc showed an apparent CPE under neutral conditions. To evaluate cell viability, the infected cells were analyzed using the viral ToxGlo assay at 8 dpi (Fig. S2). The rL showed intense CPE under low and neutral pH conditions, and cell viability decreased under both low and neutral conditions; however, syncytia formation was not observed even under low pH conditions (Fig. 2). In contrast, the cell viability of rGc-infected cells was significantly lower under the low pH conditions. Both double mutant viruses, rGn/L and rGc/L, exhibited intense CPE, and cell fusion was detected under low pH conditions at the given time points (Fig. 2). However, once a robust CPE is initiated, it is challenging to observe low pH-dependent cell fusion activity, often because of cell detachment.

### Effects of amino acid alteration on GP on the step of virus entry to Vero cells

**Fig 3:**
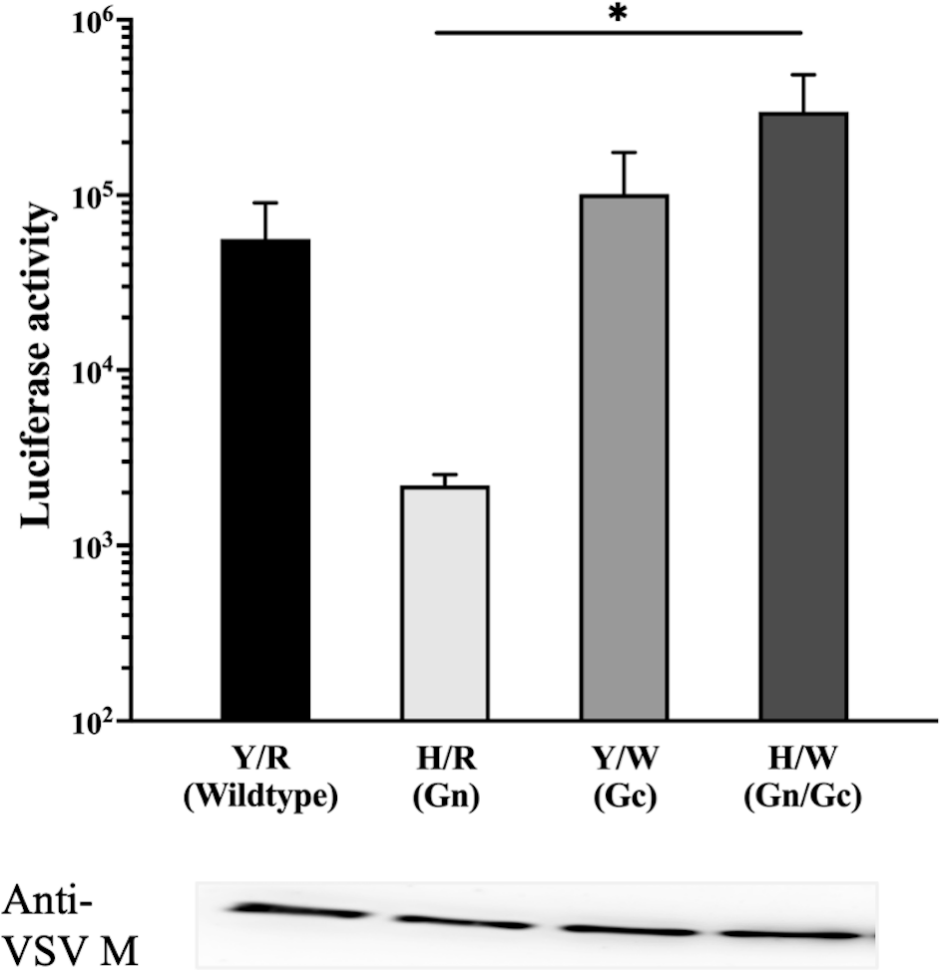
Comparison of viral entry using pseudotype viruses. Pseudotyped vesicular stomatitis viruses bearing GP-Y/R, GP-H/R, GP-Y/W, or GP-H/W instead of VSV-G were inoculated into Vero E6 cells, and luciferase activity was measured at 18 h post-inoculation. The approximate viral inoculum was evaluated by western blotting with an anti-VSV-M antibody. The results are presented as the mean and standard deviation of triplicate independent experiments. *P < 0.05.

To evaluate the efficacy of viral cell entry, we generated pseudotyped viruses bearing YG1-GP-Y/R, GP-H/R, GP-Y/W, or GP-H/W, inoculated them into Vero E6 cells, and measured the luciferase activity. The luciferase activity of VSV∆G/SFTSV-GP-H/R tended to decrease from VSV∆G/SFTSV-GP-Y/R (Fig. 3). In contrast, mutation Gc (R624W) alone did not appear to affect viral entry. The luciferase activity of VSV∆G/SFTSV-GP-H/W with both mutations (Y328H and R624W) recovered the levels shown by wild-type GP, indicating this mutation may offset the entry disadvantage caused by the Gn Y328H mutation.

### Programmed cell death related to SFTSV infection

**Fig 4:**
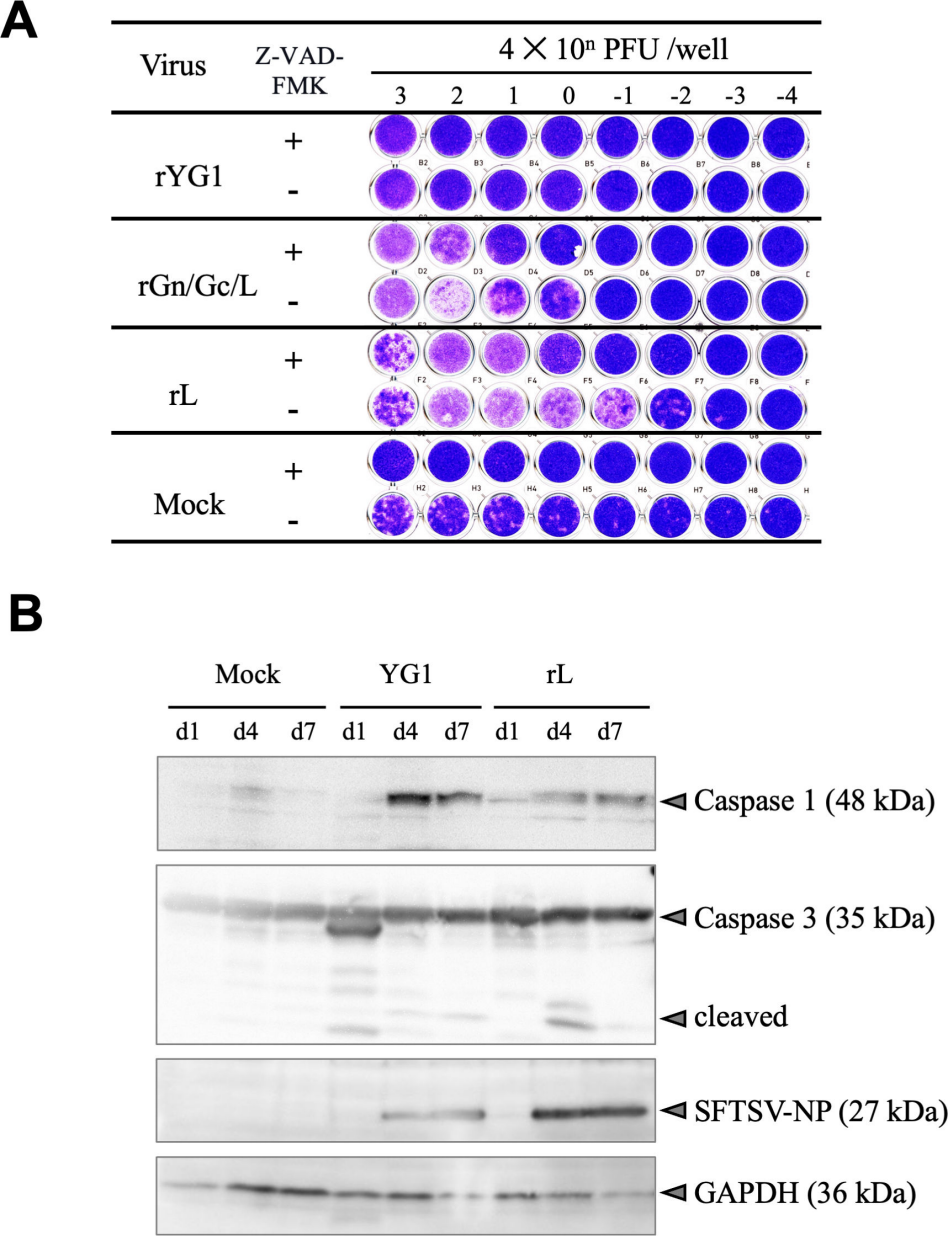
Caspase activity in SFTSV-infected cells. (A) Vero E6 cells were inoculated with recombinant viruses, and media were replaced with fresh media containing 50 µM caspase inhibitor, Z-VAD-FMK (+), or dimethyl sulfoxide alone as diluent control (−) at 3 dpi. Mock-infected Vero E6 cells were used as the controls. Five days post-inoculation, cells were fixed and stained with Giemsa azure-eosin-methylene blue solution. (B) Recombinant viruses rYG1 and rL were inoculated into Vero E6 cells. The cells were harvested at 1, 4, and 7 dpi and evaluated for protein expression using western blot analysis.

The mutation N1891K increased polymerase activity in the minigenome assay; however, there was no significant difference in growth kinetics between subclones (25). As a result, we hypothesized that the induction of CPE by subclone B7, which possesses the N1891K mutation, was not because of enhanced viral replication but was exerted via a different mechanism. To evaluate the mechanisms of CPE induction, we examined the effects of the pan-caspase inhibitor Z-VAD-FMK on rGn/Gc/L-, rL-, and rYG1-infected cells. As shown in Fig. 4A, Z-VAD-FMK reduced cell death induced by rGn/Gc/L and rL infection compared with cells without inhibitors. In contrast, rYG1 infection did not cause cell death with or without an inhibitor. These results indicated that the induction of cell death by rGn/Gc/L and rL was caspase dependent. Next, we evaluated the expression of programmed cell death-related molecules in the SFTSV-infected cells. As shown in Fig. 4B, caspase 1 and 3 induction were detected in both rL and rYG1 virus-infected cells. These results suggest that both CPE-inducing and non-inducing SFTSV infection trigger programmed cell death.

### Inhibitory effect of wild-type SFTSV on actinomycin D-induced cell death

**Fig 5:**
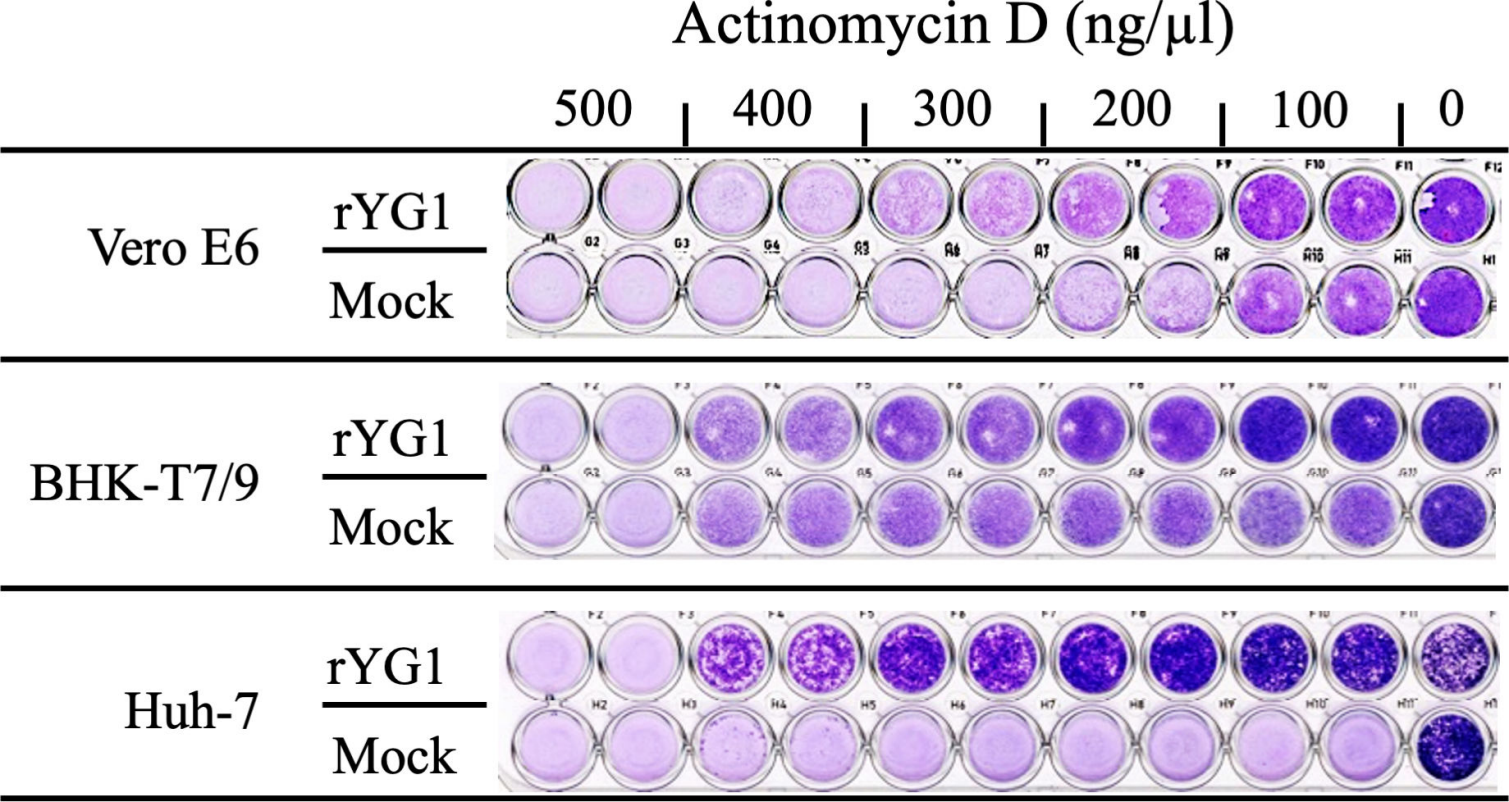
The impact of SFTSV on actinomycin D-induced cell death in cell lines. Vero E6, Huh-7, and BHK/T7-9 cell monolayers were inoculated with 10 PFU of the rYG1 virus per well. Actinomycin D in high to low concentrations (500, 400, 300, 200, and 100 ng/µL) was mixed with inoculum before inoculation. rYG1 infection alone (0 ng/mL) and actinomycin D stimulation alone (mock infection) were controls. After 3 dpi, the cells were fixed and stained with Giemsa azure-eosin-methylene blue solution for visualization.

Caspase 1 was activated in both rYG1- and rL-infected cells, with the induction of caspase 1 being more pronounced in rYG1-infected cells compared to the rL-infected cells. Nevertheless, rYG1-infected cells did not exhibit CPE in infected cells. Next, rYG1 was inoculated into cells treated with actinomycin D, a drug that induces apoptosis. As shown in Fig. 5, rYG1 infection alone did not induce cell damage. Furthermore, rYG1 inoculation reduced actinomycin D-induced cell damage. High concentrations of actinomycin D (500 ng/mL) induced cell death in both rYG1- and mock-infected cells. If the actinomycin D concentration decreased, cell death was suppressed by rYG1 infection in all the cell lines tested (Vero E6, BHK-T7/9, and Huh7 cells). These results suggest that rYG1 infection inhibited programmed cell death induced by actinomycin D. This experiment was performed 2–3 times with continuous observation. Although cells treated with actinomycin D died by 7 dpi in three cell lines, cell death was delayed in rYG1-infected cells. In Fig. 3, fixed and stained cells at 3 dpi were shown.

### Competition of wild-type and recombinant viruses in virus propagation

To compare the superiority of virus replication under coexistence conditions, we evaluated co-infection with rYG1 and recombinant viruses. Monolayered Vero E6 cells in a 6-well plate were co-inoculated with rYG1 and rGn, rGc, or rL at the respective ratios. After 1 h of incubation, each supernatant was replaced with a growth medium and incubated for 7 days. The ratio of rYG1 to recombinant viruses was estimated from the viral genome ratio in each culture supernatant (Table 2). In all combinations, rYG1 was the dominant population after co-culture with equal volumes of recombinant viruses. Even when a 10 times higher inoculation dose was inoculated, recombinant viruses were not the major population in the culture supernatant. In the 1:10 rL inoculation, the mutant virus population was reduced to 21.7% after 7 days of infection. In addition, CPE was not observed in any of the combinations.

**TABLE 2:** Viral propagation in co-infection with recombinant viruses

Virus	Altered nucleotide genome: position	Ratio wild type:mutant	Wild type %	Mutant %
rGn	M: 1000	1:10	66.7	30.8
		1:1	97.0	3.0
		1:0.1	98.0	0.0
rGc	M: 1888	1:10	60.8	38.7
		1:1	89.9	10.1
		1:0.1	99.7	0.0
rL	L: 5689	1:10	78.3	21.7
		1:1	96.4	2.7
		1:0.1	99.2	0.0

## DISCUSSION

SFTS is an emerging infectious disease with a high fatality rate caused by a member of the *Phenuiviridae* family, the *Bandavirus* genus, and has become a notable health concern in Asian countries owing to the lack of therapeutic and pathological knowledge (29–33). Several studies have reported the importance of quasispecies in the pathogenicity and growth strategies (34, 35). Understanding the characteristics of the constructs in SFTSV quasispecies may shed light on viral propagation and the pathogenicity of the population. Our previous findings showed that YG1 isolates are quasispecies and comprise subclones with distinct properties. Analysis of the identified amino acid differences in subclones demonstrated that the amino acid at position 624 in the Gc of the YG1 strain plays an essential role in low pH-dependent cell fusion activity (20). In addition, the polarity of amino acid position 1891 in the L protein of YG1 is critical for polymerase activity, and the L segment C-terminal domain is critical for genome transcription and viral replication (25). However, the role of the Y328H variation in Gn is yet to be clarified.

The Y328H variation in Gn was observed in approximately 30% of the viral population in patient blood; however, following virus isolation using Vero E6 cells, the proportion of this Gn variant decreased (26). This phenomenon may explain why SFTSV in the patient’s blood comprises approximately 70% of rYG1 and 30% of rGn, which might be pathogenic variants in this fatal infection. It is assumed that selective pressure exists in patients with the Y328H mutation in Gn leading to quasispecies formation. To detect the virological characteristics of each mutation in YG1 quasispecies *in vitro*, we recovered recombinant viruses using a newly developed reverse genetics system for the YG1 strain.

To ensure the stability of the induced mutations in the GP and L genes and to confirm the absence of unintended mutations, we performed Sanger sequencing of the GP and L mutational regions of interest in the P3 viral stock, which was used for all downstream analyses in this study. The genome sequence was confirmed by using a total RNA from P2 inoculated cells which produced P3. Given that SFTSV is an RNA virus, mutations can naturally occur during replication; however, all experiments were conducted using the same lot of P3 virus. Additionally, in the co-infection experiment, the P4 generation was sequenced using Nanopore sequencing, which revealed unexpected bases at a frequency of less than 2%. While this is likely due to the lower accuracy of Nanopore sequencing, the possibility of real mutations cannot be entirely ruled out. Notably, co-infection assays were conducted at three different ratios, and no common mutations exceeding 1% were observed across these conditions, indicating that no significant mutations occurred that would affect the experimental outcomes. These findings confirm the stability of the viral populations used in this study. However, we acknowledge the importance of further comprehensive assessments, and in future studies, we plan to perform deep sequencing across the entire viral genome to further confirm the absence of off-target changes.

Data obtained from the plaque-forming assay revealed phenotypic differences in the foci of each recombinant virus. The rGn virus with the Y328H mutation in Gn showed large plaques, suggesting that there may be differences in the virus spreading patterns from infected cells to neighboring cells in the overlay medium. Isolated subclones possessed both Gn and Gc mutations in GP, indicating that the Gn variant may have acquired R624W Gc mutations while adapting to Vero E6 cells (21). Pseudotype assay showed that the Y328H mutation in GP tended to inhibit cell entry, whereas an additional mutation in Gc (R624W) potentially mitigated this disadvantage during the entry process in Vero E6 cells. These observations suggested that the Gn variant may have acquired R624W Gc mutations during adaptation to Vero E6 cells. Several studies showed that phlebovirus Gc plays a vital role in the fusion process. In contrast, Gn probably contributes to receptor binding, depending on its position on the viral surface (36). SFTSV Gn binds to non-muscle myosin heavy chain IIA, which is critical for cellular entry of SFTSV (37). The Y328H single mutation was disadvantageous for entry into Vero E6 cells; however, it might be advantageous for unknown target cells *in vivo*.

The Gc structure of Rift Valley fever virus is similar to the class II fusion protein architecture, which has been identified as an effector of membrane fusion (38). The recombinant viruses with R624W mutations in this study showed high fusion activity. The rGc variant propagated to a titer comparable to that of rGn and rYG1 in Vero E6 cells. However, the virus with the R624W mutation alone was also disadvantageous for growth in Vero E6 cells, as evidenced by plaque-forming ability and the results of the competitive growth assay. Indeed, subclones with only the R624W mutation in Gc were not detected throughout the limiting dilution cloning process or in the patient sample. Although rGn/Gc/L induces cell death more rapidly and exhibits greater robustness than other viruses, presenting challenges in collecting viral stocks and demonstrating the lowest titers, it nevertheless forms distinct plaques, similar to rGn. These findings suggested that both mutations are essential for the efficient propagation of subclones in Vero E6 cells. Whereas the competitive growth assay indicated a preference for the growth of rYG1, which did not form clear plaques or induce CPE, compared to the other mutants. Subclone B7, whose genome sequence is identical to that of rGn/Gc/L, showed growth kinetics similar to that of the parent YG1 in Vero E6 cells. Although its proportion in the quasispecies was identified to be over ten times lower during the isolation process. This highlights the potential involvement of unknown functions or interactions with host factors in the transmission and virulence of rYG1.

In our previous study, we directly evaluated the effect of the N1891K alteration on polymerase activity using a minigenome reporter assay, which mimics the infected-cell environment. This assay involved overexpression of wild-type and mutant L proteins in T7 polymerase-expressing cells, followed by introducing a minigenome under the T7 promoter to assess polymerase activity (25). However, we could not detect reporter activity when we applied this system to SFTSV-infected T7 polymerase-transiently and stably expressing cells. We inferred that the detection sensitivity was insufficient due to the lower polymerase levels in infected cells compared to the overexpression system and challenges in infection and transfection efficiencies. These limitations suggest that while the minigenome system helps study polymerase activity in an overexpression model, its application in actual SFTSV infection may require further optimization. Future studies should explore alternative approaches to directly assess polymerase activity in the context of SFTSV infection to enhance our understanding of the functional impact of these mutations. Although alteration of N1891K in the L protein plays an essential role in CPE induction, the mechanisms underlying the induction of cell death remain unclear. A recent study suggested that the nucleotide-binding domain, leucine-rich-containing family, pyrin domain-containing-3 inflammasome, and pyroptosis are activated during SFTSV infection, leading to IL-1β/IL-18 secretion (23, 39–42). The necrotic cell death inhibitor IM-54 did not affect cell death caused by B7 or rL (data not shown). The pan-caspase inhibitor, Z-VAD-FMK, inhibited cell death induced by rL and rGn/Gc/L, suggesting a caspase-dependent mechanism (Fig. 4). However, the possibility of apoptosis induction was not confirmed, because PARP cleavage was not confirmed in rL-infected cells (data not shown). In contrast, both CPE-inducing and non-inducing viruses induced caspase 1, a pyroptosis-associated caspase. Wild-type viruses allow cells to escape death after the induction of caspase 1, whereas viruses with the N1891K mutation in the L genome segment failed to suppress cell death for unknown reasons. Suppression of cell death by SFTSV was observed during actinomycin D-induced apoptosis. Therefore, SFTSV probably acts close to the final stage of cell death, which may be involved in the pyroptosis and apoptosis cascades. Further studies are needed to clarify the mechanism of CPE induction relating to the N1891K mutation of SFTSV.

We previously reported that alteration of N1891K in the L protein induced high levels of RdRp activity (25). However, the correlation between elevated RdRp activity and induction of cell death remains unclear. One possibility is that the high production of viral RNA and proteins within the cell causes substantial stress, leading to cell death. In this study, we demonstrated another possibility that the L protein suppresses cell death, with the N1891K portion being involved in this step. However, further studies are required to clarify the mechanisms underlying the CPE caused by this alteration. Additionally, this variation N1891K may not be advantageous for viral survival in Vero E6 cells. The wild-type virus outperformed the rL virus in the competitive growth assay. Because the induction of CPE in infected cells is not considered beneficial for viral survival, SFTSV may have a mechanism to avoid cell death. When a cell is infected with a virus, the choice to produce interferons in order to be antiviral or choose cell death and eliminate itself is based on the exact mechanism mediated by the apoptosis signal-regulating kinase family kinases and is selected depending on the context (43). During SFTSV infection, non-structural protein plays a role in suppressing the interferon system (44–48). This study suggests that SFTSV may possess an additional mechanism for resisting host responses, including apoptosis.

In this study, we obtained insight into viral propagation strategies by analyzing quasispecies derived from a single fatal case. These observations suggest that the combination of mutations may enhance the viability of mutant viruses, facilitating the selection of viruses to establish a suitable population for propagation and potentially leading to the emergence of novel pathogenic viruses.

## Data Availability

The authors confirm that all data underlying the findings are fully available without restriction. All relevant data are within the paper and its supplemental material.

## SUPPLEMENTAL MATERIAL

Fig. S1 jvi.01794-24-s0001.tif Plaque diameters.

Fig. S2 jvi.01794-24-s0002.tif Viability of recombinant SFTSV-infected Vero E6 cells before and after low pH treatment.

Supplemental legends jvi.01794-24-s0003.docx Legends for Fig. S1 and S2.

ASM does not own the copyrights to Supplemental Material that may be linked to, or accessed through, an article. The authors have granted ASM a non-exclusive, world-wide license to publish the Supplemental Material files. Please contact the corresponding author directly for reuse.

## References

[1] Xu B, Liu L, Huang X, Ma H, Zhang Y, Du Y, Wang P, Tang X, Wang H, Kang K, Zhang S, Zhao G, Wu W, Yang Y, Chen H, Mu F, Chen W. 2011. Metagenomic analysis of fever, thrombocytopenia and leukopenia syndrome (FTLS) in Henan Province, China: discovery of a new bunyavirus. PLoS Pathog 7:e1002369. doi:10.1371/journal.ppat.1002369 22114553 PMC3219706

[2] Takahashi T, Maeda K, Suzuki T, Ishido A, Shigeoka T, Tominaga T, Kamei T, Honda M, Ninomiya D, Sakai T. 2014. The first identification and retrospective study of severe fever with thrombocytopenia syndrome in Japan. J Infect Dis 209:816–827. doi:10.1093/infdis/jit603 24231186 PMC7107388

[3] Kim KH, Ko MK, Kim N, Kim HH, Yi J. 2017. Seroprevalence of severe fever with thrombocytopenia syndrome in Southeastern Korea, 2015. J Korean Med Sci 32:29–32. doi:10.3346/jkms.2017.32.1.29 27914128 PMC5143294

[4] Tran XC, Yun Y, Van An L, Kim S-H, Thao NTP, Man PKC, Yoo JR, Heo ST, Cho N-H, Lee KH. 2019. Endemic severe fever with thrombocytopenia syndrome, Vietnam. Emerg Infect Dis 25:1029–1031. doi:10.3201/eid2505.181463 31002059 PMC6478219

[5] Zohaib A, Zhang J, Saqib M, Athar MA, Hussain MH, Chen J, Sial A-U-R, Tayyab MH, Batool M, Khan S. 2020. Serologic evidence of severe fever with thrombocytopenia syndrome virus and related viruses in Pakistan. Emerg Infect Dis 26:1513–1516. doi:10.3201/eid2607.190611 32568060 PMC7323538

[6] Win AM, Nguyen YTH, Kim Y, Ha NY, Kang JG, Kim H, San B, Kyaw O, Htike WW, Choi DO, Lee KH, Cho NH. 2020. Genotypic heterogeneity of Orientia tsutsugamushi in scrub typhus patients and thrombocytopenia syndrome co-infection, Myanmar. Emerg Infect Dis 26:1878–1881. doi:10.3201/eid2608.200135 32687023 PMC7392420

[7] Liu Q, He B, Huang SY, Wei F, Zhu XQ. 2014. Severe fever with thrombocytopenia syndrome, an emerging tick-borne zoonosis. Lancet Infect Dis 14:763–772. doi:10.1016/S1473-3099(14)70718-2 24837566

[8] Robles NJC, Han HJ, Park SJ, Choi YK. 2018. Epidemiology of severe fever and thrombocytopenia syndrome virus infection and the need for therapeutics for the prevention. Clin Exp Vaccine Res 7:43–50. doi:10.7774/cevr.2018.7.1.43 29399579 PMC5795044

[9] Kobayashi Y, Kato H, Yamagishi T, Shimada T, Matsui T, Yoshikawa T, Kurosu T, Shimojima M, Morikawa S, Hasegawa H, Saijo M, Oishi K, Japan S. 2020. Severe fever with thrombocytopenia syndrome, Japan, 2013-2017. Emerg Infect Dis 26:692–699. doi:10.3201/eid2604.191011 32186502 PMC7101122

[10] Mita T. 2019. Epidemiology of severe fever with thrombocytopenia syndrome in Japan. Juntendo Medical Journal 65:130–135. doi:10.14789/jmj.2019.65.JMJ18-R08

[11] Brennan B, Li P, Zhang S, Li A, Liang M, Li D, Elliott RM. 2015. Reverse genetics system for severe fever with thrombocytopenia syndrome virus. J Virol 89:3026–3037. doi:10.1128/JVI.03432-14 25552716 PMC4337530

[12] Elliott RM, Schmaljohn CS. 2013. Bunyaviridae, p 1244–1282. In Knipe DM, Howley PM (ed), Fields Virology, 6th ed. Wolters Kluwerz.

[13] Tani H. 2014. Analyses of entry mechanisms of novel emerging viruses using pseudotype VSV system. Trop Med Health 42:71–82. doi:10.2149/tmh.2014-S10 25425954 PMC4204049

[14] Plegge T, Hofmann-Winkler H, Spiegel M, Pöhlmann S. 2016. Evidence that processing of the severe fever with thrombocytopenia syndrome virus Gn/Gc polyprotein is critical for viral infectivity and requires an internal Gc signal peptide. PLoS One 11:e0166013. doi:10.1371/journal.pone.0166013 27855227 PMC5113920

[15] Hofmann H, Li X, Zhang X, Liu W, Kühl A, Kaup F, Soldan SS, González-Scarano F, Weber F, He Y, Pöhlmann S. 2013. Severe fever with thrombocytopenia virus glycoproteins are targeted by neutralizing antibodies and can use DC-SIGN as a receptor for pH-dependent entry into human and animal cell lines. J Virol 87:4384–4394. doi:10.1128/JVI.02628-12 23388721 PMC3624395

[16] Tani H, Shimojima M, Fukushi S, Yoshikawa T, Fukuma A, Taniguchi S, Morikawa S, Saijo M. 2016. Characterization of glycoprotein-mediated entry of severe fever with thrombocytopenia syndrome virus. J Virol 90:5292–5301. doi:10.1128/JVI.00110-16 26984731 PMC4934762

[17] Filone CM, Heise M, Doms RW, Bertolotti-Ciarlet A. 2006. Development and characterization of a Rift Valley fever virus cell-cell fusion assay using alphavirus replicon vectors. Virology (Auckl) 356:155–164. doi:10.1016/j.virol.2006.07.035 PMC7134558 16945399

[18] Lozach PY, Mancini R, Bitto D, Meier R, Oestereich L, Overby AK, Pettersson RF, Helenius A. 2010. Entry of bunyaviruses into mammalian cells. Cell Host Microbe 7:488–499. doi:10.1016/j.chom.2010.05.007 20542252 PMC7172475

[19] Tani H, Kawachi K, Kimura M, Taniguchi S, Shimojima M, Fukushi S, Igarashi M, Morikawa S, Saijo M. 2019. Identification of the amino acid residue important for fusion of severe fever with thrombocytopenia syndrome virus glycoprotein. Virology (Auckl) 535:102–110. doi:10.1016/j.virol.2019.06.014 31299486

[20] Tsuda Y, Igarashi M, Ito R, Nishio S, Shimizu K, Yoshimatsu K, Arikawa J. 2017. The amino acid at position 624 in the glycoprotein of SFTSV (severe fever with thrombocytopenia virus) plays a critical role in low-pH-dependent cell fusion activity. Biomed Res 38:89–97. doi:10.2220/biomedres.38.89 28442665

[21] Nishio S, Tsuda Y, Ito R, Shimizu K, Yoshimatsu K, Arikawa J. 2017. Establishment of subclones of the severe fever with thrombocytopenia syndrome virus YG1 strain selected using low pH-dependent cell fusion activity. Jpn J Infect Dis 70:388–393. doi:10.7883/yoken.JJID.2016.357 28003599

[22] Gowen BB, Westover JB, Miao J, Van Wettere AJ, Rigas JD, Hickerson BT, Jung K-H, Li R, Conrad BL, Nielson S, Furuta Y, Wang Z. 2017. Modeling severe fever with thrombocytopenia syndrome virus infection in golden syrian hamsters: importance of STAT2 in preventing disease and effective treatment with Favipiravir. J Virol 91:e01942-16. doi:10.1128/JVI.01942-16 27881648 PMC5244333

[23] Gao C, Yu Y, Wen C, Li Z, Ding H, Qi X, Cardona CJ, Xing Z. 2022. Nonstructural protein NSs activates inflammasome and pyroptosis through interaction with NLRP3 in human microglial cells infected with severe fever with thrombocytopenia syndrome bandavirus. J Virol 96:e0016722. doi:10.1128/jvi.00167-22 35695505 PMC9278151

[24] Suzuki T, Sato Y, Sano K, Arashiro T, Katano H, Nakajima N, Shimojima M, Kataoka M, Takahashi K, Wada Y, Morikawa S, Fukushi S, Yoshikawa T, Saijo M, Hasegawa H. 2020. Severe fever with thrombocytopenia syndrome virus targets B cells in lethal human infections. J Clin Invest 130:799–812. doi:10.1172/JCI129171 31904586 PMC6994144

[25] Noda K, Tsuda Y, Kozawa F, Igarashi M, Shimizu K, Arikawa J, Yoshimatsu K. 2020. The polarity of an amino acid at position 1891 of severe fever with thrombocytopenia syndrome virus L protein is critical for the polymerase activity. Viruses 13:33. doi:10.3390/v13010033 33375489 PMC7823514

[26] Yoshikawa T, Shimojima M, Fukushi S, Tani H, Fukuma A, Taniguchi S, Singh H, Suda Y, Shirabe K, Toda S. 2015. Phylogenetic and geographic relationships of severe fever with thrombocytopenia syndrome virus in China, South Korea, and Japan. J Infect Dis 212:889–898. doi:10.1093/infdis/jiv144 25762790

[27] Buchholz UJ, Finke S, Conzelmann KK. 1999. Generation of bovine respiratory syncytial virus (BRSV) from cDNA: BRSV NS2 is not essential for virus replication in tissue culture, and the human RSV leader region acts as a functional BRSV genome promoter. J Virol 73:251–259. doi:10.1128/JVI.73.1.251-259.1999 9847328 PMC103829

[28] Lundu T, Tsuda Y, Ito R, Shimizu K, Kobayashi S, Yoshii K, Yoshimatsu K, Arikawa J, Kariwa H. 2018. Targeting of severe fever with thrombocytopenia syndrome virus structural proteins to the ERGIC (endoplasmic reticulum Golgi intermediate compartment) and Golgi complex. Biomed Res 39:27–38. doi:10.2220/biomedres.39.27 29467349

[29] Miyauchi A, Sada KE, Yamamoto H, Iriyoshi H, Touyama Y, Hashimoto D, Nojima S, Yamanaka S, Ishijima K, Maeda K, Kawamura M. 2022. Suspected transmission of severe fever with thrombocytopenia syndrome virus from a cat to a veterinarian by a single contact: a case report. Viruses 14:223. doi:10.3390/v14020223 35215817 PMC8874511

[30] Yu X-J, Liang M-F, Zhang S-Y, Liu Y, Li J-D, Sun Y-L, Zhang L, Zhang Q-F, Popov VL, Li C. 2011. Fever with thrombocytopenia associated with a novel bunyavirus in China. N Engl J Med 364:1523–1532. doi:10.1056/NEJMoa1010095 21410387 PMC3113718

[31] Gong Z, Gu S, Zhang Y, Sun J, Wu X, Ling F, Shi W, Zhang P, Li D, Mao H, Zhang L, Wen D, Zhou B, Zhang H, Huang Y, Zhang R, Jiang J, Lin J, Xia S, Chen E, Chen Z. 2015. Probable aerosol transmission of severe fever with thrombocytopenia syndrome virus in southeastern China. Clin Microbiol Infect 21:1115–1120. doi:10.1016/j.cmi.2015.07.024 26255811

[32] Choi SJ, Park SW, Bae IG, Kim SH, Ryu SY, Kim HA, Jang HC, Hur J, Jun JB, Jung Y, Chang HH, Kim YK, Yi J, Kim KH, Hwang JH, Kim YS, Jeong HW, Song KH, Park WB, Kim ES, Oh MD, Korea SCN. 2016. Severe fever with thrombocytopenia syndrome in South Korea, 2013-2015. PLoS Negl Trop Dis 10:e0005264. doi:10.1371/journal.pntd.0005264 28033338 PMC5226827

[33] Kato H, Yamagishi T, Shimada T, Matsui T, Shimojima M, Saijo M, Oishi K, SFTS epidemiological research group-Japan. 2016. Epidemiological and clinical features of severe fever with thrombocytopenia syndrome in Japan, 2013-2014. PLoS ONE 11:e0165207. doi:10.1371/journal.pone.0165207 27776187 PMC5077122

[34] Grande-Pérez A, Martin V, Moreno H, Torre JC. 2015. Edited by E. Domingo and P. Schuster. Quasispecies: from theory to experimental systems. current topics in microbiology and immunology. Springer Cham.

[35] Sevilla N, de la Torre JC. 2006. Arenavirus diversity and evolution: quasispecies in vivo. Curr Top Microbiol Immunol 299:315–335. doi:10.1007/3-540-26397-7_11 16568904 PMC7120374

[36] Wu Y, Zhu Y, Gao F, Jiao Y, Oladejo BO, Chai Y, Bi Y, Lu S, Dong M, Zhang C, Huang G, Wong G, Li N, Zhang Y, Li Y, Feng WH, Shi Y, Liang M, Zhang R, Qi J, Gao GF. 2017. Structures of phlebovirus glycoprotein Gn and identification of a neutralizing antibody epitope. Proc Natl Acad Sci USA 114:E7564–E7573. doi:10.1073/pnas.1705176114 28827346 PMC5594662

[37] Sun Y, Qi Y, Liu C, Gao W, Chen P, Fu L, Peng B, Wang H, Jing Z, Zhong G, Li W. 2014. Nonmuscle myosin heavy chain IIA is a critical factor contributing to the efficiency of early infection of severe fever with thrombocytopenia syndrome virus. J Virol 88:237–248. doi:10.1128/JVI.02141-13 24155382 PMC3911693

[38] Dessau M, Modis Y. 2013. Crystal structure of glycoprotein C from Rift Valley fever virus. Proc Natl Acad Sci USA 110:1696–1701. doi:10.1073/pnas.1217780110 23319635 PMC3562824

[39] Li Z, Hu J, Bao C, Gao C, Zhang N, Cardona CJ, Xing Z. 2022. Activation of the NLRP3 inflammasome and elevation of interleukin-1β secretion in infection by sever fever with thrombocytopenia syndrome virus. Sci Rep 12:2573. doi:10.1038/s41598-022-06229-0 35173184 PMC8850576

[40] Liu JW, Chu M, Jiao YJ, Zhou CM, Qi R, Yu XJ. 2021. SFTSV infection induced interleukin-1β secretion through NLRP3 inflammasome activation. Front Immunol 12:595140. doi:10.3389/fimmu.2021.595140 33708197 PMC7940371

[41] Li S, Li H, Zhang YL, Xin QL, Guan ZQ, Chen X, Zhang XA, Li XK, Xiao GF, Lozach PY, Cui J, Liu W, Zhang LK, Peng K. 2020. SFTSV infection induces BAK/BAX-dependent mitochondrial DNA release to trigger NLRP3 inflammasome activation. Cell Rep 30:4370–4385. doi:10.1016/j.celrep.2020.02.105 32234474

[42] Yu S, Zhang Q, Su L, He J, Shi W, Yan H, Mao H, Sun Y, Cheng D, Wang X, Zhang Y, Fang L. 2023. Dabie bandavirus infection induces macrophagic pyroptosis and this process is attenuated by platelets. PLoS Negl Trop Dis 17:e0011488. doi:10.1371/journal.pntd.0011488 37486928 PMC10399884

[43] Okazaki T, Higuchi M, Takeda K, Iwatsuki-Horimoto K, Kiso M, Miyagishi M, Yanai H, Kato A, Yoneyama M, Fujita T, Taniguchi T, Kawaoka Y, Ichijo H, Gotoh Y. 2015. The ASK family kinases differentially mediate induction of type I interferon and apoptosis during the antiviral response. Sci Signal 8. doi:10.1126/scisignal.aab1883 26243192

[44] Zhang L, Fu Y, Zhang R, Guan Y, Jiang N, Zheng N, Wu Z. 2021. Nonstructural protein NSs hampers cellular antiviral response through LSm14A during severe fever with thrombocytopenia syndrome virus infection. J Immunol 207:590–601. doi:10.4049/jimmunol.2100148 34244294

[45] Zhang S, Zheng B, Wang T, Li A, Wan J, Qu J, Li CH, Li D, Liang M. 2017. NSs protein of severe fever with thrombocytopenia syndrome virus suppresses interferon production through different mechanism than Rift Valley fever virus. Acta Virol 61:289–298. doi:10.4149/av_2017_307 28854793

[46] Yoshikawa R, Kawakami M, Yasuda J. 2023. The NSs protein of severe fever with thrombocytopenia syndrome virus differentially inhibits the type 1 interferon response among animal species. J Biol Chem 299:104819. doi:10.1016/j.jbc.2023.104819 37187292 PMC10276162

[47] Wu X, Qi X, Qu B, Zhang Z, Liang M, Li C, Cardona CJ, Li D, Xing Z. 2014. Evasion of antiviral immunity through sequestering of TBK1/IKKε/IRF3 into viral inclusion bodies. J Virol 88:3067–3076. doi:10.1128/JVI.03510-13 24335286 PMC3957960

[48] Wuerth JD, Weber F. 2016. Phleboviruses and the type I interferon response. Viruses 8:174. doi:10.3390/v8060174 27338447 PMC4926194

