# Supplementary figures and images for: Characterization of quasispecies of severe fever with thrombocytopenia syndrome virus
Source: J Virol. 2025 Apr 9;99(5):e01794-24. doi: 10.1128/jvi.01794-24 (PMC12090785; doi:10.1128/jvi.01794-24)

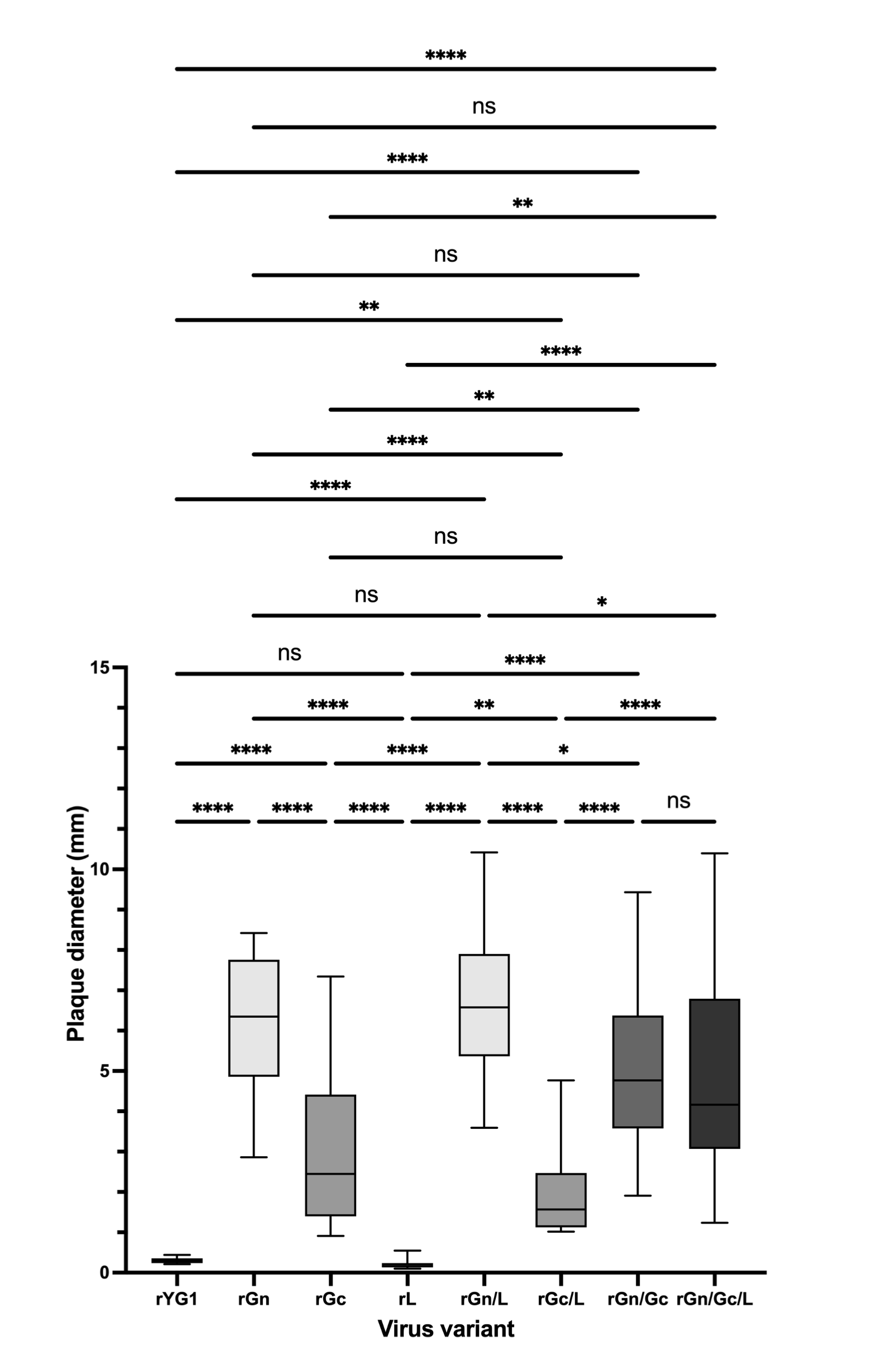

Supplement: Fig. S1 — Plaque diameters. [file jvi.01794-24-s0001.tif]

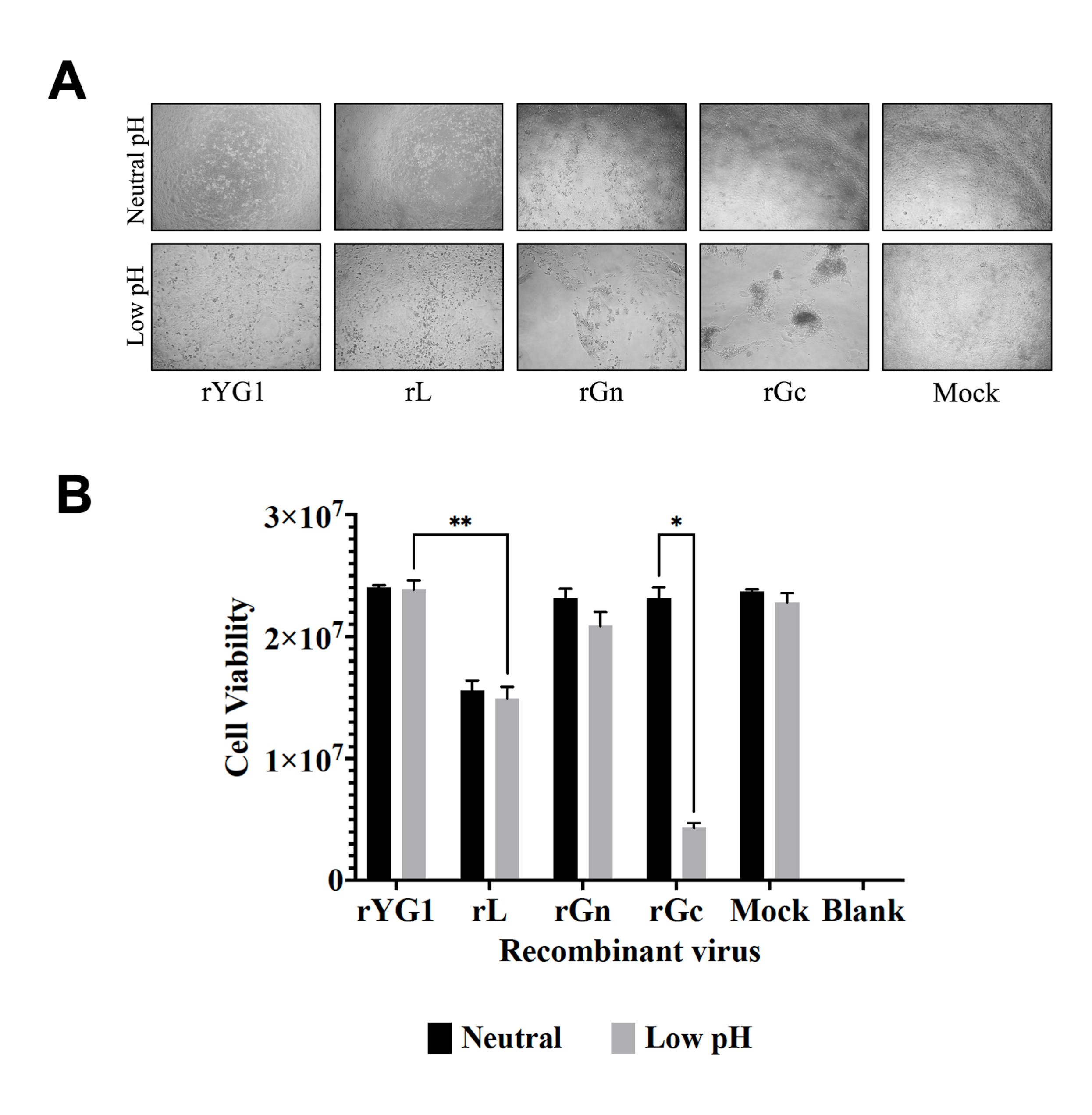

Supplement: Fig. S2 — Viability of recombinant SFTSV-infected Vero E6 cells before and after low pH treatment. [file jvi.01794-24-s0002.tif]
