# Supplementary material for: Characterization of quasispecies of severe fever with thrombocytopenia syndrome virus
Source: J Virol. 2025 Apr 9;99(5):e01794-24. doi: 10.1128/jvi.01794-24 (PMC12090785; doi:10.1128/jvi.01794-24)
Supplement: Supplemental legends — Legends for Fig. S1 and S2. [file jvi.01794-24-s0003.docx]

Supplemental Figure 1: The plaque diameters.

Box-and-whisker plots show the diameter of randomly selected 25 plaques of each recombinant virus where whiskers represent minimum to maximum data points. The plaque diameters of all combinations shown in Fig. 1b were compared and the results of statistical analysis are presented. ns: *P* > 0.05, **P* < 0.05, ** *P* < 0.01, **** *P* < 0.0001.

Supplemental Figure 2: Viability of recombinant SFTSV-infected Vero E6 cells before and after low pH treatment.

The Vero E6 cells were seeded in 96 well plates at 1x10^4^ cells and achieved 80-90% confluency 18-24 hours after seeding. After 18 hours of incubation, cells were infected with the recombinant viruses; rYG1, rL, rGn, rGc, and the Mock at 1x10^2^ PFU/well. At seven dpi, cells were treated with 50 mM acetate-buffered saline (pH 5.6) and incubated for 2 min at room temperature. Then, the medium was replaced with fresh growth medium and incubated for 24 h at 37 °C. Images were taken the following day, and a CPE assay was conducted using Viral ToxGlo (Promega Corporation, Madison, WI, USA) according to the manufacturer’s protocols. The 100 μl of ATP Detection Reagent was added to each well of the 96-well plate. Then, 50 μl of lysate was added to a 96-well half-area white flat-bottom plate (Corning Incorporated, NY, USA). The luminescence was measured using the GloMax Multi Detection System (Promega) after incubating for 10 min at room temperature. Raw luminescence was plotted versus each recombinant virus, and the unpaired T-test was calculated using GraphPad prism. * *P* < 0.05, ** *P* < 0.001.
